# Microparticles Produced by Activated Platelets Carry a Potent and Functionally Active Angiogenic Signal in Subjects with Crohn’s Disease

**DOI:** 10.3390/ijms19102921

**Published:** 2018-09-26

**Authors:** Eleonora Gaetani, Fabio Del Zompo, Margherita Marcantoni, Ilaria Gatto, Igor Giarretta, Angelo Porfidia, Franco Scaldaferri, Lucrezia Laterza, Loris Lopetuso, Antonio Gasbarrini, Roberto Pola

**Affiliations:** 1 Division of Internal Medicine and Gastroenterology, Department of Medicine, Fondazione Policlinico Universitario Agostino Gemelli IRCCS, Università Cattolica del Sacro Cuore, 00168 Rome, Italy; eleonora.gaetani@unicatt.it (E.G.); fabio.delzompo@gmail.com (F.D.Z.); franco.scaldaferri@policlinicogemelli.it (F.S.); laterza.lucrezia@gmail.com (L.L.); lopetusoloris@libero.it (L.L.); antonio.gasbarrini@unicatt.it (A.G.); 2 Division of Vascular Medicine, Department of Medicine, Fondazione Policlinico Universitario Agostino Gemelli IRCCS, Università Cattolica del Sacro Cuore, 00168 Rome, Italy; margheritamarcantoni@yahoo.it (M.M.); ilariagatto@yahoo.it (I.G.); igor.giarretta@unicatt.it (I.G.); angelo.porfidia@policlinicogemelli.it (A.P.)

**Keywords:** microparticles, angiogenesis, Crohn’s disease

## Abstract

Microparticles (MPs) are submicron vesicles shed from various cell types upon activation, stimulation, and death. Activated platelets are an important source of circulating MPs in subjects with inflammatory diseases, including Crohn’s disease (CD). Angiogenesis is a hallmark of inflammation in CD and plays an active role in sustaining disease progression, while targeting angiogenesis may be an effective approach to block colitis. In this study, we analyzed the angiogenic content of the MPs produced by activated platelets in subjects with CD. We also evaluated whether the angiogenic signal carried by these MPs was functionally active, or able to induce angiogenesis. We found that, in subjects with CD, MPs produced by activated platelets contain significantly higher levels of angiogenic mRNAs, such as epidermal growth factor (EGF), platelet-derived growth factor-α (PDGFα), fibroblast growth factor (FGF-2), and angiopoietin-1 (ANGPT1), compared to MPs isolated from control subjects. They also contain significantly higher levels of prototypical angiogenic proteins, including vascular endothelial growth factor (VEGF), angiopoietin-1, endoglin, endothelin-1, pentraxin 3, platelet factor-4, plasminogen activator inhibitor-1 (PAI-1), tissue inhibitor of metalloproteinases-1 (TIMP-1), and thrombospondin 1. The protein content of these MPs is functionally active, since it has the ability to induce a robust angiogenic process in an endothelial cell/interstitial cell co-culture in vitro assay. Our results reveal a potential novel mechanism through which the angiogenic signal is delivered in subjects with CD, with potentially important clinical and therapeutic implications.

## 1. Introduction

Microparticles (MPs) are a type of extracellular vesicle produced by cells upon activation, stimulation, and death, through budding of the plasma membrane, formation of membrane “blebs”, and eventual release into the bloodstream [1]. MPs are between 100 and 1000 nm in size and bear on their surface the antigenic markers of their ancestral cell [2]. Although once considered to be just inert remnants of cellular processes, MPs are now recognized as important players in many physiological and pathological states, due to the fact that they contain important biological information, including nucleic acids and proteins, and are able to act as vectors for the transfer of biological messages from producing to target cells [1].

Platelet-derived MPs comprise the vast majority of circulating MPs (up to 70–90%), even in healthy individuals [3]. Their production and release in the circulation increases significantly upon activation of platelets by thrombin, calcium, collagen, complement, and shear forces [4]. In recent years, a number of studies have demonstrated higher numbers of circulating MPs in subjects with inflammatory diseases, including Crohn’s disease (CD) [5,6,7,8]. However, these studies have mainly focused on the quantification of MPs and the analysis of certain specific MP functional properties, such as the ability to bind annexin. No attention has been paid to the biological information contained in MPs in the setting of CD.

Angiogenesis is a hallmark of inflammation in CD and plays an active role in sustaining disease progression [9], while targeting angiogenesis may be an effective approach to block colitis [10,11]. Previous studies have shown that MPs produced by platelets may contribute to the angiogenic process in the setting of tumor development and metastasis, as well as in cardiovascular diseases [12,13]. It has been hypothesized that this occurs by fusion of MPs with target cells and transfer of pro-angiogenic growth factors, modulators, and second messengers [13].

In this study, we analyzed the number and angiogenic content of MPs produced by activated platelets (aPMPs) circulating in the bloodstream of subjects with CD. We found that aPMPs are increased in the plasma of subjects with active CD (aCD), compared to healthy subjects (HS) and subjects with inactive CD (iCD), and their number correlates with the diseases’ activity and C-reactive protein (CRP) levels. In addition, in subjects with aCD, aPMPs carry high levels of angiogenic mRNAs and proteins. This content is functionally active, since it is able to induce angiogenesis in vitro.

## 2. Results

### 2.1. Subjects with aCD Have an Increased Number of Circulating aPMPs

The demographic and clinical characteristics of the studied population are presented in Table 1. The three groups (aCD, iCD, and HS) were matched in terms of age and sex. The Crohn’s Disease Activity Index (CDAI) was 316.9 ± 89.4 in subjects with aCD and 105 ± 64.1 in subjects with iCD (*p* < 0.01). CRP was significantly higher in the aCD group compared to both subjects with iCD and HS (*p* < 0.01).

**Figure 1:**
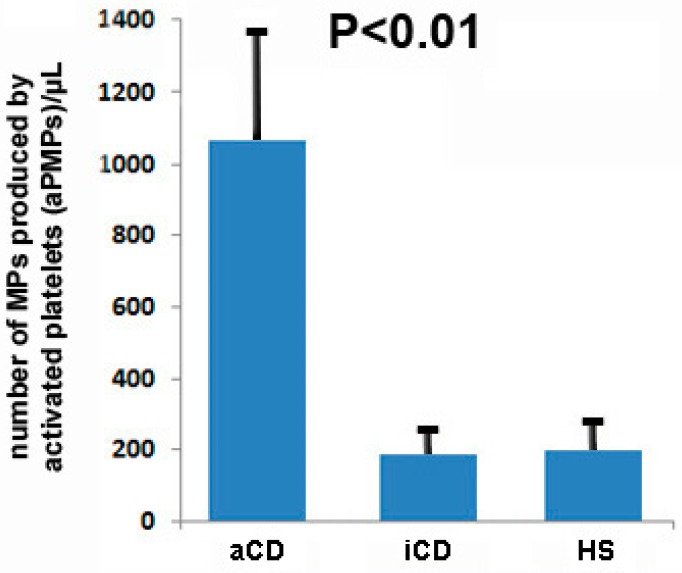
Number of microparticles (MPs), produced by activated platelets, detected by means of cytofluorimetry, in the blood of subjects with active Crohn’s disease (aCD), inactive Crohn’s disease (iCD), and healthy subjects (HS).

Figure 1 presents the number of MPs produced by activated platelets, detected by the means of cytofluorimetry, in the blood of HS and subjects with aCD and iCD. The number of circulating aPMPs (CD62P+) was significantly higher in patients with aCD than in individuals with iCD and HS (1064.7 ± 331.2/µL, 189.8 ± 38.4/µL, and 200.7 ± 44.7/µL, respectively, *p* < 0.01).

**Figure 2:**
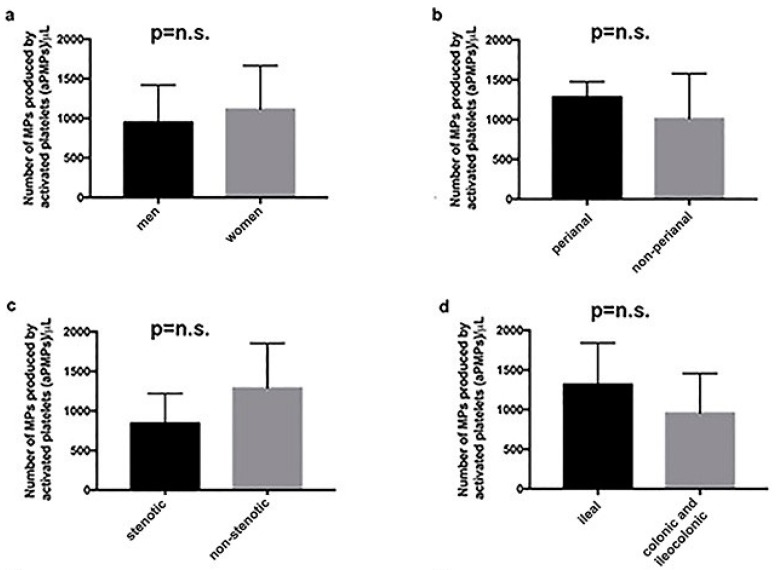

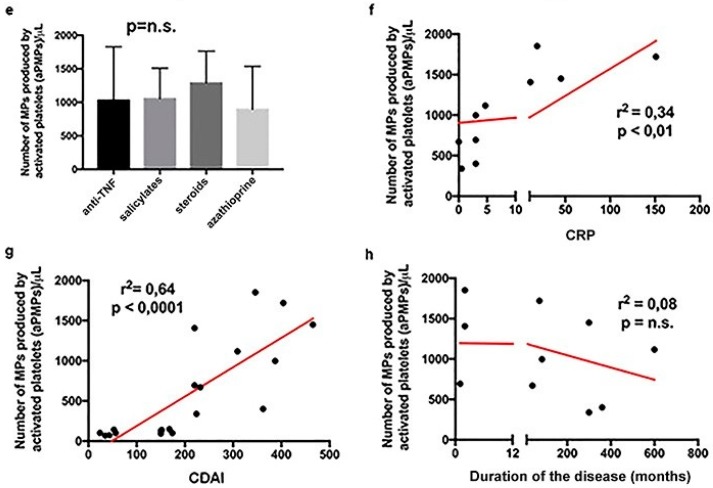
Among subjects with aCD, the number of aPMP is not affected by gender (**a**), presence or absence of perianal disease (**b**), stenotic disease (**c**), ileal or colonic disease (**d**), and type of treatment (**e**). Instead, it correlated with C-reactive protein (CRP) levels (**f**) and Crohn’s Disease Activity Index (CDAI) (**g**). Finally, it does not depend on the duration of the disease (**h**). n.s.: not significant. In panels (**f**–**h**), the black points are actual samples, connected with linear regression by a single, straight, red line.

In the group of individuals with aCD, the number of aPMPs was similar among men and women (Figure 2a), as well as among subjects with perianal and non-perianal disease (Figure 2b), subjects with the stenotic and the inflammatory form of the disease (Figure 2c), and subjects with only ileal localization or colonic/ileocolonic localization of the disease (Figure 2d). Likewise, the number of circulating aPMPs was not different among subjects with aCD treated with either steroids, salicylates, azathioprine, or anti-TNF mAb (Figure 2e). On the other hand, there was a significant correlation between the number of circulating aPMPs and the plasma levels of CRP (*p* < 0.01) (Figure 2f). A highly significant association also existed between the number of circulating aPMPs and CDAI (*p* < 0.0001) (Figure 2g). Instead, the number of circulating aPMPs was not influenced by the duration of the disease (Figure 2h).

### 2.2. Circulating aPMPs Are Rich in Angiogenic mRNAs and Proteins in Subjects with aCD

To determine the angiogenic content of aPMPs, we first used an angiogenic-specific PCR array, able to assess the expression levels of 84 prototypical angiogenesis-related mRNAs. This analysis was carried out on a discovery cohort of 7 patients with aCD and 7 HS. The expression of 12 mRNAs was significantly different between subjects with aCD and HS (Table 2). In particular, the following 10 mRNAs were significantly upregulated in aCD subjects: Epidermal growth factor (EGF), Fibroblast growth factor 2 (FGF-2), Insulin-like growth factor 1 (IGF-1), Platelet-derived growth factor α (PDGFα), Angiopoietin 1 (ANGPT1), Matrix metallopeptidase 2 (MMP2), Matrix metallopeptidase 9 (MMP9), Fibronectin 1 (FN1), Integrin α V (ITGAV), and Integrin beta 3 (ITGB3). The following two mRNAs were instead down-regulated in aCD subjects compared to HS: Serpin peptidase inhibitor F (SERPINF1) and Vascular endothelial growth factor B (VEGF-B).

To further validate these findings, we performed RT-PCRs targeted to the genes of interest. This validation analysis was carried out on all the 40 aCD individuals and the 40 HS enrolled in the study. It led to the confirmation of significantly higher content of the following mRNAs in the aPMPs of subjects with aCD, compared to HS: EGF, PDGFα, FGF-2, and ANGPT1 (Table 3).

We also looked at the angiogenic content of aPMPs at the protein level. This analysis was performed on a sample of 7 individuals with aCD and 7 HS, using a human angiogenesis antibody array, which allows to simultaneously determine the levels of 55 prototypical angiogenesis-related proteins. Circulating aPMPs of subjects with aCD contained significantly higher levels of the following proteins, compared to controls: Angiogenin (Ang), Platelet factor-4 (PF4), SERPINF1, Plasminogen activator inhibitor-1 (PAI-1), Metallopeptidase inhibitor 1 (TIMP-1), and Thrombospondin 1 (TSP-1) (Table 4).

We also found angiogenic proteins that were contained exclusively in the aPMPs of subjects with aCD and were undetectable in HS. Table 5 presents the list of the proteins that were detected in ≥4 out of 7 aCD patients and in 0 out of 7 HS. This list includes crucial angiogenic molecules, such as VEGF, angiopoietin-1, EGF, endoglin, endothelin-1, PDGF-AB, PDGF-AA, and pentraxin 3 (PTX3).

### 2.3. Proteins Extracted from aPMPs of Subjects with aCD Have the Ability to Promote Angiogenesis

**Figure 3:**
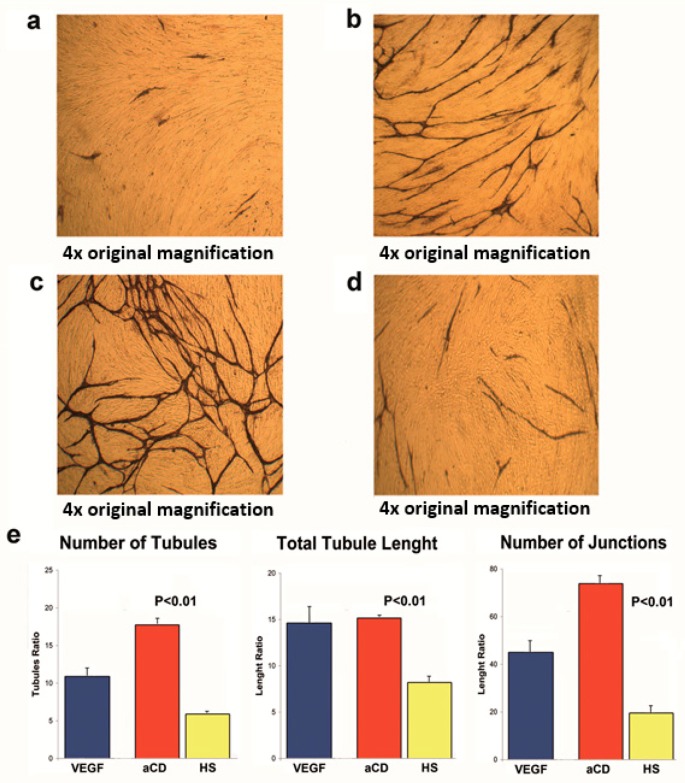
Endothelial/interstitial cells’ co-culture assay showing formation of tubule-like structures in the absence of stimulating factors (**a**) and upon addition of VEGF; (**b**) protein extract of aPMPs of subjects with aCD (**c**), and protein extract of aPMPs of HS (**d**). Quantification analyses of tubule-like structures in terms of number of tubules, tubule length, and number of junctions (**e**).

For the study of angiogenesis, we used a co-culture system containing both endothelial cells and interstitial cells. In this assay, endothelial cells have the tendency to spontaneously form tubule-like, CD31-positive structures (Figure 3a). The addition of the angiogenic cytokine VEGF led to an increase of tubule formation (Figure 3b). Similarly, the addition of the protein extract of aPMPs of subjects with aCD greatly increased tubule formation (Figure 3c). In contrast, the addition of the protein extract of aPMPs of HS did not result in a similar effect (Figure 3d). Quantification analyses demonstrated that the angiogenic properties of the protein extract of aPMPs of subjects with aCD were significantly greater than those of HS in terms of the number of tubules, tubule length, and number of junctions (Figure 2e).

## 3. Discussion

There are only a limited number of studies that have analyzed circulating MPs in subjects with CD. Chamouard et al. reported an increased overall number of circulating MPs in CD patients compared to healthy subjects [5]. Other authors found an increased number of MPs produced by activated platelets in the circulation of subjects with CD [6,7,8,14]. Our findings consisted of these data and confirmed an elevated number of circulating aPMPs in subjects with aCD, while clinical remission of the disease was associated with a number of circulating aPMPs that were similar to that observed in healthy individuals. Chamouard et al. also reported that anti-TNFα treatment reduces the number of circulating MPs in subjects with CD [5]. In our study, the number of circulating aPMPs was not different among subjects treated with anti-TNFα mAbs, steroids, salicylates, or azathioprine. Instead, it correlated with disease activity (as measured by CDAI) and severity of inflammation (as measured by CRP levels). This suggests that the number of circulating aPMPs does not depend on the type of treatment, but on the severity and activity level of the disease.

To our knowledge, ours is also the first study that demonstrates that the aPMPs that circulate in the plasma of subjects with aCD are rich in angiogenic RNAs and proteins, and that the proteins contained in these MPs are functionally active, since they have the ability to induce a strong angiogenic process in vitro. In terms of functional significance of circulating MPs in CD, only one study is available in the medical literature, and it reports that the intravenous injection of MPs in mice isolated from humans with CD has a potent effect on endothelial function and vascular reactivity [7].

We have identified four angiogenic mRNAs that are significantly upregulated in the aPMPs of subjects with aCD: EGF, PDGFα, FGF-2, and ANGPT1. This was intriguing, since the EGF pathway is among those considered dysregulated in IBD [15], PDGFα is expressed, along with its receptors, in areas of active IBD inflammation [16], FGF-2 is high in the serum of subjects with CD and correlates with bowel wall thickness [17], and ANGPT1 serum levels decrease upon treatment with anti-TNFα in CD patients who achieve remission [18]. Regarding the angiogenic proteins that we have identified, the concentration of some of them is higher in the MPs produced by the activated platelets of subjects with aCD than in those produced by the activated platelets of HS, while others are detectable only in the aPMPs of aCD individuals and not in the aPMPs of HS. Among these proteins, some deserve to be mentioned for their established or possible role in CD pathobiology. One is Angiogenin, which is known to be significantly higher in the serum of IBD patients compared to healthy subjects [19]. Another is endothelin-1, which was reported to be elevated in CD mucosal samples 24 years ago already [20]. Finally, particularly interesting was the detection of PTX3 exclusively in the aPMPs of aCD subjects (5 out of 7) and not in those of HS (0 out of 7). Indeed, PTX3 has been recently proposed as a novel marker of CD, as it appears to be superior to CRP in predicting CD activity [21]. This is based on the findings that PTX3 serum levels are significantly increased in patients with aCD compared with patients in remission, its expression is higher in inflamed colonic tissues compared with uninflamed colonic tissues, and its serum levels positively correlate with disease activity in CD. Taken together, these findings suggest that aPMPs are vectors of functionally relevant biological messages in the setting of bowel inflammation.

Strong confirmation of this hypothesis is provided by the results of our angiogenic assay, which has demonstrated that a very robust angiogenic process may be induced in vitro using the protein content of aPMPs isolated from the blood of aCD subjects. This angiogenic process has morphological characteristics that are different from the angiogenesis induced by VEGF in vivo, probably because it is the result of the action of multiple molecules and not only of VEGF. This is an important consideration with potentially relevant therapeutic implications, as it suggests that efficient anti-angiogenic therapies in CD may not be obtained by simply contrasting VEGF, but instead require targeted inhibition of multiple molecules. In addition, anti-angiogenic therapies should also take into account the possibility that, in CD, the angiogenic message may be carried through the body by circulating vectors, such as MPs.

Our findings are consistent with previous studies that have shown that MPs produced by platelets have the ability to promote angiogenesis. Indeed, platelet-derived MPs have long been known to increase in the circulation of subjects with cancer and have been implicated in various steps of cancer development and progression, including shrouding of circulating tumor cells, immune evasion, induction of a procoagulant state associated with increased risk for venous thromboembolism, and establishment of niches for anchorage of circulating tumor cells [3,12]. It has also been shown that treatment with PMPs results in increased angiogenesis at the infarct boundary zone in an experimental model of stroke in rats [22], and that intravenous injection of PMPs improves neovascularization in a rodent model of hindlimb ischemia [23]. Finally, the results of our in vitro studies are consistent with previous findings by Anene et al., who have found that co-culturing PMPs with human umbilical vein endothelial cells (HUVEC) on extracellular matrix gel induces a robust, capillary-like structure formation, with significantly reduced production of the anti-angiogenic agent, thrombospondin-1 (THBS-1), from endothelial cells [13].

Our study has some limitations. In particular, it remains to be elucidated how important the contribution of the signal contained in aPMPs is to angiogenesis and mucosal inflammation in humans with CD. However, this was beyond the aims of our study, and further investigations are needed to address this issue. An additional limitation is that we did not analyze the microRNA content of MPs. MicroRNAs are an important regulator of gene expression and their role in CD is under intensive investigation [13]. Angiogenic microRNAs are known to be shuttled by MPs and are considered novel relevant players in cardiovascular diseases [24,25]. Their presence and role in the MPs of CD patients deserves investigation in additional studies.

## 4. Materials and Methods

### 4.1. Patients

Patients with CD were recruited among those presenting to the Department of Medicine, Division of Gastroenterology, of the Fondazione Policlinico Universitario Agostino Gemelli IRCCS of Rome, Italy, between Febuary 2013 and December 2017. The diagnosis of CD was done using clinical, biochemical, radiological, endoscopic, and histological parameters. The disease was considered active according to the Lennard-Jones criteria, and clinical activity was quantified using the CD Activity Index (CDAI) [26,27], which takes into account the following: (i) the number of liquid or soft stools each day for seven days; (ii) abdominal pain (graded from 0–3 on severity) each day for seven days; (iii) general wellbeing, subjectively assessed from 0 (well) to 4 (terrible) each day for seven days; (iv) presence of an abdominal mass at clinical examination (0 as none, 2 as questionable, 5 as definite); (v) taking drugs for diarrhea; (vi) hematocrit of <0.47 in men and <0.42 in women; (vii) percentage deviation from standard weight; (ix) and the presence of the following complications: arthralgia, uveitis, erythema nodosum, aphtous ulcers in mouth, anal fissure, anal fistula, and abscess. As established, the disease was considered active when CDAI was >220. It was instead considered in clinical remission when CDAI was <150 or when there was clinical and biochemical evidence of disease inactivity and CDAI was between 150 and 220. Exclusion criteria from the study were: current or recent (within one month) intestinal infection or extra-digestive infection, pregnancy, or cancer. At the end of the recruitment period, 40 subjects with aCD and 30 subjects with iCD were enrolled. We also enrolled a control group of 40 healthy subjects (HS) without a history of gastrointestinal diseases or familial history of digestive disorders, especially a history of inflammatory bowel disease in particular. Venous blood was collected from all enrolled subjects for the determination of the following: total white cell count, polymorphonuclear leukocytes, lymphocytes, platelet count, hemoglobin, CRP, and albumin. All subjects gave their informed consent for inclusion before they participated in the study. The study was conducted in accordance with the Declaration of Helsinki, and the protocol was approved by the Ethics Committee of the Fondazione Policlinico Universitario Agostino Gemelli IRCCS/Università Cattolica del Sacro Cuore (protocol number P/491/CE/2011, approved on 19 May 2011).

### 4.2. Isolation and Characterization of aPMPs

Blood samples were drawn by sterile venous puncture and collected into citrate vacutainer tubes for flow cytometry (3 mL) and into EDTA tubes for the other analysis (12 mL). All samples were processed immediately after blood collection. Platelet-rich plasma (PRP) was obtained by centrifugation at 450× *g* for 20 min at room temperature. The supernatant was immediately further centrifuged at 1500× *g* for 20 min to generate platelet-free plasma (PFP) and processed within 2 h for flow-cytometry acquisition. PFP was diluted 1:3 with cold PBS, and MPs were collected by an ultra-centrifugation step at 100,000× *g* for 80 s at 4 °C for the remaining experiments. MP types were characterized according to the expression of membrane-specific antigens by flow cytometry. For the identification of aPMPs, 50 µL of PFP were incubated for 30 min with 1 µL of fluorescein isothiocyanate (FITC)-labeled anti-CD42b antibody (Beckman Coulter, Brea, CA, USA) and phycoerythrin (PE)-labeled anti-CD62P antibody (Beckman Coulter). An equal volume of fluorospheres was added to samples in order to determine the MP number, and analyzed by a FC500 Flow Cytometer (Beckman Coulter). The fluorescent Megamix beads (Biocytex, Marseille, France) covering the MPs (0.5 and 0.9 µm) and platelet-size ranges (0.9 and 3 µm) allowed for standardization of the set-up of MPs’ analysis regions and to get reproducible MP counts. A total of 250,000 events were acquired for each sample; values are reported as the number of MPs per µL of platelet-free plasma (number/µL).

### 4.3. Angiogenic mRNA Signature of Circulating aPMPs

Cell-sorting was used to isolate aPMPs from the blood of HS and individuals with aCD. RNA was extracted from MP pellets with a Trizol (Invitrogen, Carlsbad, CA, USA)-based method. Then, it was resuspended in 30 µL of DNA/RNAse-free water and treated with 6U of DNAse I (Qiagen, Hilden, Germany) to eliminate genomic DNA contamination. Then, it was purified on a column using the RNeasy micro kit (Qiagen), according to the manufacturer’s instructions. RNA quantity and quality were assessed with the Nanodrop 2000 (Thermo Scientific, Waltham, MA, USA) and the Agilent Bioanalyzer RNA Pico Chip (Agilent, Santa Clara, CA, USA). Because of the low amount of starting RNA, 20 ng of RNA from each sample were reverse-transcribed and 5 µL of cDNA were pre-amplified using the RT² PreAMP cDNA Synthesis Kit (Qiagen), according to the manufacturer’s recommendations. Gene-expression profiling was performed using the RT² Profiler PCR Array for the Angiogenesis Pathway (Qiagen; PAHS-024Z) in a discovery cohort of 7 subjects with aCD and 7 HS. The amplification was performed using a CFX- 96 (Biorad, Hercules, CA, USA) with the following protocol: 95 °C, 10 min; 40 cycles of 95 °C, 15 s; and 60 °C, 1 min. The relative level of mRNAs expression was calculated using the ΔΔCT method and analyzed with the RT^2^ Profiler PCR Array Data Analysis Template v4.0 (Qiagen, Hilden, Germany). The results of these angiogenic arrays were validated by RT-PCRs targeted to the genes of interest. The validation cohort consisted of 40 aCD individuals and 40 HS.

### 4.4. Analysis of Angiogenic Proteins Contained in Circulating aPMPs

Circulating aPMPs were isolated by means of cell-sorting from the blood of HS (*n* = 7) and individuals with aCD (*n* = 7). MP pellets were lysed with RIPA buffer (Tris-HCl 50 mM; NaCl 150 mM; SDS 0.1%; Na-deoxycolate 0.5%; Tryton 1%). Extracted proteins were quantified by using the Bradford method (Biorad). For each subject, 100 µg of proteins were analyzed by using a human angiogenesis antibody array (R&D Systems, Minneapolis, MN, USA), which allowed for the simultaneous detection of 55 human angiogenesis-related proteins. All samples were analyzed in duplicate. The arrays were scanned with the Chemidoc XRS System (Biorad) and analyzed with the Lab Image Software 4.0 (Biorad).

### 4.5. Endothelial Cell/Interstitial Cell Co-Culture Assay

We used a commercially available assay (TCS CellWorks, Buckingham, UK), in which proliferating early passage normal human endothelial cells are co-cultured with early passage normal human interstitial cells in 24-well plates, in a specially formulated culture medium. In this assay, cells spontaneously proliferate and then enter a migratory phase, during which they move through the matrix to form, after 11 days in culture, a network of capillary-like tubules [28]. This degree of spontaneous angiogenic activity was evaluated and used as a control to quantify the angiogenic effects induced by the addition of proteins extracted by aPMPs of HS (*n* = 7) and individuals with aCD (*n* = 7). Proteins were added at a concentration of 10 nmol/L. VEGF was used as a positive control at a concentration of 10 ng/mL, as suggested by the manufacturer (TCS CellWorks). On day 11, cells were fixed with ice-cold 70% ethanol, and tubule formation was visualized by immunostaining for the endothelial cell marker CD31 and quantified by image analysis (TCS AngioSys software, CellWorks, Buckingham, UK), as previously described [11]. The number of tubules, total tubule length, and number of junctions were calculated in untreated cells and in cells stimulated with proteins extracted from aPMPs of HS and CD subjects and VEGF. All of the experiments were performed in duplicate. Results were expressed as a ratio between the angiogenic effect induced by either proteins extracted from aPMPs and VEGF and that observed in untreated cells. All quantifications were performed in a blinded fashion by two independent operators.

### 4.6. Statistical Analysis

Differences between groups in Table 1 were analyzed either by a one-way analysis of variance (ANOVA) or *X*-square test. The Mann—Whitney U-test was used to compare the number of circulating microparticles between groups (Figure 1 and Figure 2a–e). A linear regression analysis was done to evaluate the correlation between microparticles and CRP levels, CDAI, and disease duration (Figure 2f–h). ANOVA was used to analyze differences between groups in the in vitro assays. A *p* value < 0.05 was considered to be statistically significant.

## Figures and Tables

**Table 1:** Demographical and clinical characteristics of the studied population.

Demographical and Clinical Characteristics	aCD	iCD	HS	*p*
Gender (men/women)	16/24	13/17	18/22	n.s.
Age (mean ± SD)	44.1 ± 8.4	43.8 ± 7.9	45.1 ± 5.3	n.s.
CDAI (mean ± SD)	318.9 ± 95.3	105.5 ± 64.1	–	<0.01
Disease duration in months (mean ± SD)	185.1 ± 102.5	151.6 ± 193.5	–	n.s.
Type of disease (inflammatory/stenotic)	26/14	17/13	–	n.s
Extension of disease (only ileal/colonic or ileocolonic)	10/30	7/23	–	n.s.
Perianal disease (yes/no)	9/31	5/25	–	n.s.
Treatment (steroids/salicylates/azathioprine/anti-TNF)	24/4/8/8	3/3/3/30	–	n.s.
C-reactive protein (CRP) (mg/L)	31.8 ± 56.7	1.6 ± 1.8	0.9 ± 0.8	<0.01
Total leukocytes (×1000/mcl)	7.3 ± 3.2	6.5 ± 2.1	6.8 ± 1.5	n.s.
Polymorphonuclear leukocytes (×1000/mcl)	5.4 ± 3.4	3.7 ± 1.8	4.5 ± 1.9	n.s.
Lymphocytes (×1000/mcl)	1.5 ± 0.9	1.9 ± 0.7	1.7 ± 0.5	n.s.
Platelets (×1000/mcl)	335.7 ± 152.2	256.8 ± 51.8	318.1 ± 88.5	n.s.
Hemoglobin (g/dL)	10.8 ± 2.3	12.8 ± 1.7	13.8 ± 1.6	<0.05

n.s.: Not significant.

**Table 2:** Activated platelets (aPMPs)-angiogenic mRNAs with significant fold changes between subjects with aCD and healthy controls (discovery cohort).

mRNA	Fold-Change	*p*
EGF	+2.4	0.01
FGF-2	+4.9	0.006
IGF-1	+4.0	0.02
PDGFα	+2.0	0.03
ANGPT1	+2.9	0.03
MMP2	+14.2	0.02
MMP9	+6.12	0.04
FN1	+5.6	0.04
ITGAV	+6.5	0.04
ITGB3	+3.5	0.02
SERPINF1	−4.3	0.04
VEGF-B	−5.5	0.01

**Table 3:** aPMPs-angiogenic mRNAs with significant fold changes between subjects with aCD and healthy controls (validation cohort).

mRNA	Fold-Change	*p*
EGF	+3.2	0.01
FGF-2	+5.4	0.004
PDGFα	+3.2	0.01
ANGPT1	+2.8	0.01

**Table 4:** Angiogenic proteins significantly more expressed in the aPMPs of subjects with aCD compared to healthy controls.

Protein	Fold-Change	*p*
Angiogenin	+4.2	0.005
Platelet factor-4	+4.4	0.005
SERPINF1	+3.8	0.01
Plasminogen activator inhibitor-1	+3.5	0.01
Metallopeptidase inhibitor 1	+3.1	0.01
Thrombospondin 1	+4.2	0.01

**Table 5:** Angiogenic proteins detected in the aPMPs of ≥4 (out of 7) subjects with aCD and 0 (out of 7) healthy controls (HS).

Protein	aCD Subjects	HS
Angiopoietin 1	++++/−−−	−−−−−−−
EGF	++++/−−−	−−−−−−−
Endoglin	++++/−−−	−−−−−−−
Endothelin-1	+++++/−−	−−−−−−−
IGFBP-1	++++/−−−	−−−−−−−
IGFBP-2	++++/−−−	−−−−−−−
IGFBP-3	++++/−−−	−−−−−−−
TGFB1	++++/−−−	−−−−−−−
MMP8	++++/−−−	−−−−−−−
MMP9	++++/−−−	−−−−−−−
Pentraxin 3	+++++/−−	−−−−−−−
VEGF-A	+++++/−−	−−−−−−−
PDGF-AA	++++/−−−	−−−−−−−
PDGF-AB	++++/−−−	−−−−−−−
